# Lesion Diameter Versus Inflammatory Biomarkers and Clinical Severity in Predicting Treatment Selection in Idiopathic Granulomatous Mastitis

**DOI:** 10.1002/wjs.70554

**Published:** 2026-08-31

**Authors:** Muhammet Fatih Keyif, Sabahattin Destek

**Affiliations:** ^1^ Department of General Surgery Bolu Abant Izzet Baysal University Faculty of Medicine Bolu Türkiye; ^2^ Department of General Surgery Usküdar University Faculty of Medicine İstanbul Türkiye

**Keywords:** C‐reactive protein, disease severity, idiopathic granulomatous mastitis, lesion diameter, neutrophil‐to‐lymphocyte ratio, treatment selection

## Abstract

**Background:**

Idiopathic granulomatous mastitis (IGM) is a rare chronic inflammatory breast disease with no established predictor to guide medical versus surgical treatment. Neutrophil‐to‐lymphocyte ratio (NLR) and C‐reactive protein (CRP) have been proposed, but prior reports rarely adjusted for lesion morphology or other markers of disease severity. We evaluated whether NLR, CRP, lesion diameter and clinical severity variables independently predict treatment selection in IGM.

**Methods:**

Single‐center retrospective cohort of 71 women with biopsy‐proven IGM (2015–2019). An age‐matched control group (*n* = 42) was selected from 74 healthy women. Pretreatment leukocyte, NLR, CRP and ultrasonographic lesion diameter were analyzed together with clinical severity variables (fistula/ulcer, multi‐quadrant involvement and axillary lymphadenopathy). Primary outcome was treatment modality (any surgery vs. medical only). Univariable and multivariable logistic regression were performed with an a priori biomarker‐focused model and an extended model incorporating severity variables. Sensitivity analyses assessed drainage‐only and excisional‐only subgroups. ROC analysis used bootstrap confidence intervals with pairwise DeLong tests.

**Results:**

Surgical treatment was performed in 38 patients (53.5%; 30 drainage, 8 excisional); 33 (46.5%) received medical treatment only. After age matching, leukocyte, NLR and CRP were higher in patients than controls (all *p* < 0.001; large effect sizes). On univariable analysis, only lesion diameter predicted surgical treatment (OR 1.21 per mm; *p* < 0.001). In the biomarker‐focused multivariable model, only lesion diameter remained independent (aOR 1.23; 95% CI 1.11–1.36; *p* < 0.001). In the extended model, lesion diameter retained independence (aOR 1.25; 95% CI 1.13–1.40; *p* < 0.001), whereas severity variables and biomarkers did not contribute; axillary lymphadenopathy showed an inverse association (aOR 0.08; *p* = 0.046), likely reflecting confounding with lesion size. Sensitivity analyses confirmed the predictive value of lesion diameter. Lesion diameter discriminated treatment selection with high accuracy (AUC 0.914; 22 mm cut‐off: sensitivity 92.1%, specificity 84.8%); the extended model did not improve on this (AUC 0.918; ΔAUC +0.004; DeLong *p* = 0.89).

**Conclusion:**

Inflammatory biomarkers and standard severity variables did not add independent predictive value beyond ultrasonographic lesion diameter for the medical‐versus‐surgical decision in IGM. NLR and CRP nonetheless correlated with lesion burden and may retain a role as markers of inflammatory activity rather than as independent selectors of treatment. These findings should be interpreted as predictors of treatment selection under the studied decision‐making pattern rather than as intrinsic markers of disease severity.

## 1 Introduction

Idiopathic granulomatous mastitis (IGM) is a rare benign chronic inflammatory disease of the breast that affects predominantly young, multiparous, premenopausal women with a reported prevalence concentrated in Middle Eastern, Mediterranean and Asian populations [1, 2]. Clinically and radiologically, IGM frequently mimics breast carcinoma, contributing to diagnostic delay and patient anxiety (Figure 1) [3]. The disease follows a chronic relapsing course; recurrence rates of 16%–50% have been reported despite a range of medical and surgical interventions [2, 4]. Definitive diagnosis requires histopathological demonstration of non caseating granulomas after exclusion of specific causes such as tuberculosis, sarcoidosis and corynebacterial infection [1, 5].

**FIGURE 1:**
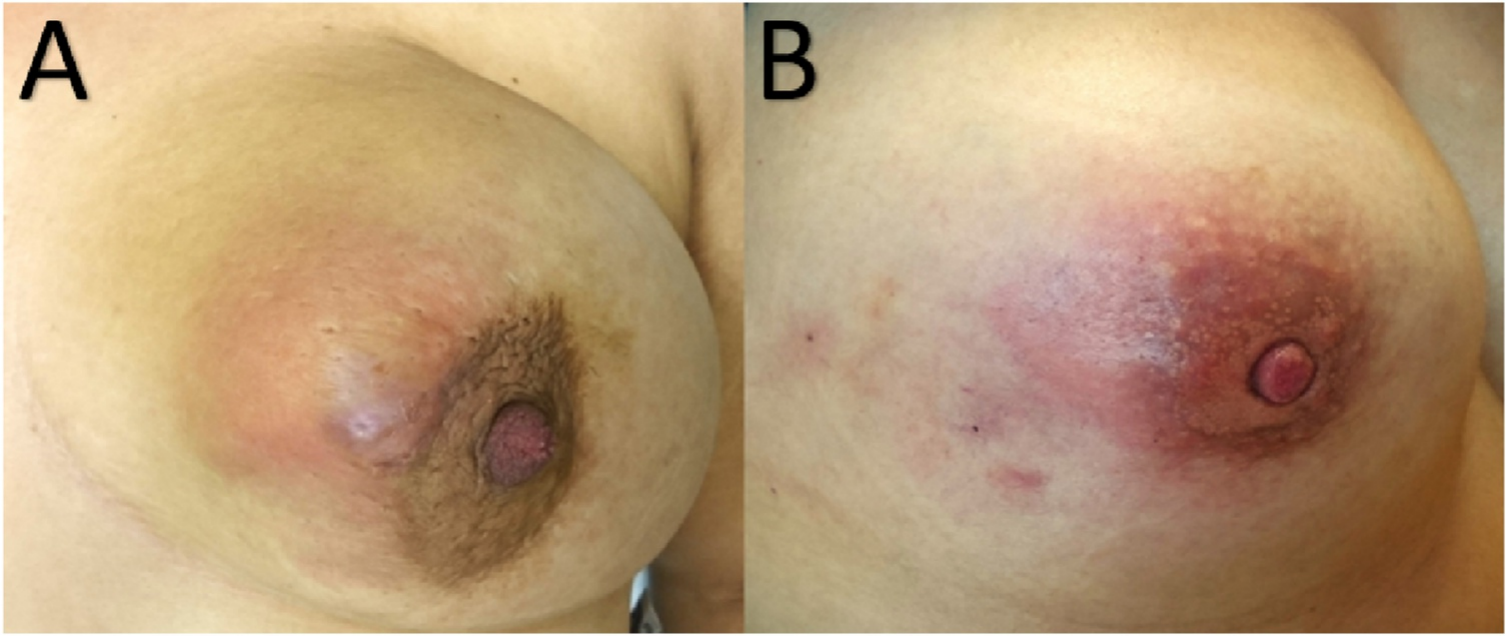
Clinical presentations of idiopathic granulomatous mastitis. (A) Periareolar erythema with nipple retraction in the left breast, illustrating the mimicry of malignant disease. (B) Widespread cutaneous erythema and induration of the right breast during the acute inflammatory phase.

The optimal treatment of IGM remains controversial. Observation, antibiotics, topical and systemic corticosteroids, methotrexate, abscess drainage, wide local excision and segmental mastectomy have all been employed, with substantial heterogeneity across centers and no universally accepted algorithm [4, 6, 7]. Surgical intervention is often selected for patients with abscess formation, fistula, treatment resistant disease or larger lesions, but the criteria for this decision are largely subjective and operator dependent.

In the search for objective predictors, peripheral inflammatory biomarkers have attracted increasing attention. The neutrophil‐to‐lymphocyte ratio (NLR) and C‐reactive protein (CRP) are inexpensive, widely available and reflect systemic inflammatory activity. Recent reports have proposed NLR as a prognostic marker for IGM recurrence [8, 9] and as a discriminator between IGM and breast cancer or bacterial mastitis [10, 11]. However, two critical gaps persist in the literature. First, most studies have examined diagnostic differentiation or recurrence prediction; the role of NLR and CRP specifically in the choice between medical and surgical treatment has not been systematically evaluated. Second, prior reports have relied largely on univariable comparisons or methodologically incomplete multivariable models, rarely incorporating lesion morphology (a clinically dominant variable) into the predictive framework. Whether inflammatory biomarkers carry independent predictive value once lesion size is accounted for therefore remains unknown.

The present study addresses this gap. We hypothesized that NLR, CRP, lesion diameter and standard clinical severity variables would each contribute independently to the prediction of surgical treatment in IGM. Using a retrospective cohort of histopathologically confirmed IGM patients and an age matched healthy control group, we performed univariable and multivariable logistic regression, ROC analysis with bootstrap validated confidence intervals and pairwise AUC comparisons to evaluate the relative and incremental predictive value of these three candidate markers.

## 2 Methods

### 2.1 Study Design and Population

This retrospective cohort study was conducted at the Department of General Surgery, Bezmialem Vakıf University Faculty of Medicine. The medical records of 92 women with a clinical and radiological preliminary diagnosis of IGM presenting between January 2015 and December 2019 were reviewed. Twenty one patients were excluded because of follow‐up at another institution, incomplete records, or alternative diagnoses on histopathology (tuberculous mastitis or sarcoidosis of the breast). The final analytic cohort comprised 71 patients with biopsy proven IGM (Supporting Information S1: Figure S1, study flow diagram).

The study was approved by the Bezmialem Vakıf University Faculty of Medicine Ethics Committee (decision number 16/299, 2019). The research was conducted in accordance with the Declaration of Helsinki. Reporting follows the STROBE guideline for observational studies.

### 2.2 Control Group

To enable patient control comparisons of inflammatory biomarkers free from age related confounding, a control group of 74 healthy women undergoing elective inguinal hernia surgery during the same period was assembled. None had a history of breast disease, autoimmune disorder, active infection or systemic inflammatory condition. Because the mean age of this initial control pool was higher than the patient group (48.7 ± 15.7 vs. 37.0 ± 8.8 years), we restricted the primary analysis to the age matched subset (ages 18–50 years; *n* = 42; mean age 37.4 ± 8.9 years), which did not differ from the patient group in age (*p* = 0.825) or BMI (*p* = 0.718). Sensitivity analyses using the full control cohort (*n* = 74) are presented in supplementary material.

### 2.3 Data Collection

Demographic, clinical, radiological, microbiological, histopathological, laboratory and treatment data were extracted from electronic medical records by two reviewers. Recorded variables included age, body mass index (BMI), Charlson Comorbidity Index (CCI), parity, breastfeeding duration, menopausal status, hormonal therapy history, comorbidities, presenting symptoms, side and quadrant of breast involvement, presence of erythema nodosum, ultrasonographic lesion diameter, axillary lymphadenopathy, mammographic and MRI findings (when performed), microbiological cultures, histopathological diagnosis and details of medical and surgical treatment.

All patients underwent breast ultrasonography. Mammography was performed in women aged ≥ 40 years and breast MRI was obtained when malignancy could not be radiologically excluded. Histopathological confirmation was achieved by ultrasound guided tru cut biopsy in all patients. Ziehl‐Neelsen, periodic acid‐Schiff and Gram stains were performed; bacterial, fungal and tuberculosis cultures were obtained.

Ultrasonographic lesion diameter was recorded as the maximum diameter of the dominant lesion as reported in the initial radiology report. In patients with multifocal disease, the maximum diameter of the largest single lesion (rather than cumulative diameter across foci) was used, consistent with the way the treating surgeon interpreted the imaging in daily practice. When multiple lesion diameters were reported for the same patient over time, the pretreatment measurement obtained at initial presentation was used.

Pretreatment laboratory parameters analyzed were serum leukocyte count (reference range 4.6–10.2 × 10^3^/μL), NLR (calculated from absolute neutrophil and lymphocyte counts) and CRP (reference range 0–5 mg/L), all measured before initiation of treatment.

#### 2.3.1 Treatment Definitions and Outcomes

Patients were grouped according to the treatment received: medical treatment only (oral or topical corticosteroids and/or antibiotics, no surgical procedure; *n* = 33) versus surgical treatment (any operative intervention—drainage, wide local excision, or segmentectomy—with or without adjunctive medical therapy; *n* = 38). The primary outcome was treatment modality (medical vs. surgical). The secondary outcome was disease recurrence during follow‐up, defined as reappearance of clinical or radiological IGM after initial resolution.

Because the study was retrospective, no protocolized decision algorithm dictated the choice between medical and surgical treatment. Treatment was determined by the attending surgeon based on clinical judgment in each case. Reviewing the clinical documentation retrospectively, drainage was typically selected when an abscess or fluctuant collection was identified clinically or radiologically; wide local excision was performed for well‐circumscribed lesions considered surgically resectable at initial presentation or after failure of medical therapy; and segmentectomy was reserved for patients with disease extending across a single breast segment in whom local excision was judged inadequate. Medical treatment (oral or topical corticosteroids with or without antibiotics) was preferred as the first‐line approach for lesions that were not fluctuant, not fistulized and not amenable to complete local excision. Although these tendencies could be reconstructed from the records, we did not have access to a written institutional protocol; the retrospective nature of this reconstruction is acknowledged as a limitation.

### 2.4 Statistical Analysis

Continuous variables are presented as mean ± SD for normally distributed data and median (interquartile range, IQR) for non‐normally distributed data; categorical variables as *n* (%). Distributional assumptions were evaluated by the Shapiro–Wilk test. Group comparisons used Student's t‐test or the Mann–Whitney *U* test for continuous variables and chi‐square or Fisher's exact test for categorical variables. Effect sizes for non‐parametric comparisons were reported as Cliff's delta.

Univariable and multivariable logistic regression analyses were performed to identify independent predictors of surgical treatment. The multivariable model included age, NLR, CRP and lesion diameter, selected a priori on clinical grounds rather than data driven selection. Model performance was assessed by Nagelkerke pseudo‐R^2^, the Hosmer–Lemeshow goodness‐of‐fit test and Akaike information criterion (AIC).

To address the possibility that additional clinical severity markers might carry independent predictive information beyond inflammatory biomarkers and lesion morphology, we constructed an extended multivariable model incorporating fistula/ulcer formation, multi‐quadrant breast involvement and axillary lymphadenopathy alongside the variables of the a priori biomarker‐focused model (age, NLR, CRP and lesion diameter). Three severity variables (bilateral disease, nipple retraction and erythema nodosum) were considered but not entered into the multivariable model because of low event counts (*n* = 13, 3 and 4 respectively) that would have precluded stable coefficient estimation. Ultrasonographic malignancy suspicion was collinear with other severity markers on preliminary examination and was retained only in the univariable analysis.

Two sensitivity analyses were performed to explore whether the observed predictors reflected surgical treatment as a whole or were driven primarily by one surgical subgroup. The first restricted the surgical group to drainage procedures (*n* = 30) versus medical treatment (*n* = 33). The second restricted the surgical group to excisional procedures (wide local excision and segmentectomy combined (*n* = 8)) versus medical treatment. Given the limited number of events in the excisional subgroup, only parsimonious multivariable models were fitted for this analysis.

Receiver operating characteristic (ROC) analysis was used to evaluate the discriminative performance of each biomarker and of biomarker combinations for surgical treatment. Optimal cut‐off values were determined by the Youden index. Bootstrap resampling with 1000 iterations was used to generate 95% confidence intervals for the area under the curve (AUC). Pairwise AUC comparisons were performed using DeLong's test for correlated ROC curves.

Recurrence analyses were prespecified as exploratory given the small number of events (*n* = 12) and reported with caution regarding limited statistical power.

Two‐sided *p*‐values < 0.05 were considered statistically significant. Analyses were performed in Python 3.11 using statsmodels, scikit‐learn and SciPy libraries; cross validated against the original SPSS analyses.

## 3 Results

### 3.1 Demographic and Clinical Characteristics

The 71 patients had a mean age of 37.0 ± 8.8 years (range 15–66) and a mean BMI of 27.0 ± 5.2 kg/m^2^. Most were premenopausal (84.5%), multiparous (93.0% with at least one delivery) and had a mean lifetime breastfeeding duration of 21.1 ± 16.3 months. Eighteen patients (25.4%) were obese (BMI ≥ 30 kg/m^2^). Charlson Comorbidity Index was 0 in 70.4%; the most frequent comorbidity was thyroiditis (16.9%). Nineteen patients (26.8%) reported any hormonal exposure, of whom IVF treatment was the most common (*n* = 10) and oral contraceptive use accounted for eight. Detailed characteristics are presented in Table 1.

**TABLE 1:** Demographic and baseline characteristics of patients with idiopathic granulomatous mastitis (*n* = 71).

Characteristic	Value
Age, years, mean ± SD (range)	37.0 ± 8.8 (15–66)
BMI, kg/m^2^, mean ± SD (range)	27.0 ± 5.2 (16.2–40.7)
Obesity (BMI ≥ 30), *n* (%)	18 (25.4)
Charlson comorbidity index, *n* (%)	
0	50 (70.4)
1	15 (21.1)
≥ 2	6 (8.5)
Common comorbidities, *n* (%)	
Thyroiditis	12 (16.9)
Inflammatory joint disease	4 (5.6)
Diabetes mellitus	3 (4.2)
Menopausal status, *n* (%)	
Premenopausal	60 (84.5)
Menopausal	7 (9.9)
Postmenopausal	4 (5.6)
Parity, *n* (%)	
0	5 (7.0)
1–2	41 (57.7)
≥ 3	25 (35.2)
Breastfeeding duration, months, mean ± SD (range)	21.1 ± 16.3 (0–72)
Hormonal exposure, *n* (%)	
None	52 (73.2)
Oral contraceptives	8 (11.3)
IVF Treatment	10 (14.1)
Hormone replacement	1 (1.4)

Abbreviations: BMI, body mass index; IVF, in vitro fertilization.

The most frequent presentation was swelling combined with pain and erythema (50.7%). Right sided involvement was most common (42.3%), followed by left (39.4%) and bilateral (18.3%); single quadrant disease was seen in 71.8% of patients. The mean ultrasonographic lesion diameter was 27.5 ± 15.2 mm (range 7–84). Axillary lymphadenopathy was observed in 28.2%, and erythema nodosum in 5.6%. Microbiological cultures from biopsy specimens were sterile in all patients (Table 2).

**TABLE 2:** Clinical and radiological features (*n* = 71).

Feature	n (%)
Presenting symptoms	
Swelling only	18 (25.4)
Swelling + pain + erythema	36 (50.7)
Swelling + pain + fistula/ulcer	14 (19.7)
With nipple retraction	3 (4.2)
Affected side	
Right	30 (42.3)
Left	28 (39.4)
Bilateral	13 (18.3)
Multiple quadrant involvement	20 (28.2)
Axillary lymphadenopathy on US	20 (28.2)
Erythema nodosum	4 (5.6)
Lesion diameter, mm, mean ± SD (range)	27.5 ± 15.2 (7–84)
Treatment	
Medical only	33 (46.5)
Any surgical procedure	38 (53.5)
Drainage	30 (42.3)
Wide local excision	5 (7.0)
Segmentectomy	3 (4.2)
Recurrence during follow‐up	12 (16.9)
Mean follow‐up, months ± SD	15.7 ± 4.3

Abbreviation: US, ultrasonography.

### 3.2 Treatment and Follow‐Up

Thirty three patients (46.5%) received medical treatment only; 38 (53.5%) underwent at least one surgical procedure. Among surgical interventions, drainage (*n* = 30, 42.3%) was most frequent, followed by wide local excision (*n* = 5, 7.0%) and segmentectomy (*n* = 3, 4.2%). Oral prednisolone was administered to 51 patients (71.8%) at a daily dose ranging from 10 to 60 mg over 4–15 weeks. The mean follow‐up duration was 15.7 ± 4.3 months, during which recurrence occurred in 12 patients (16.9%).

Median ultrasonographic lesion diameter differed markedly across treatment categories: 16.0 mm (IQR 13.0–20.0) in the medical‐only group, 33.5 mm (IQR 27.2–40.0) in the drainage group, 20.0 mm across the wide local excision patients (*n* = 5) and 49.0 mm across the segmentectomy patients (*n* = 3). These distributions are consistent with a treatment decision anchored primarily on lesion size and the presence of abscess or fluctuant collection, reinforcing the rationale for the subsequent analytical framework.

### 3.3 Inflammatory Biomarkers in Patients Versus Controls

Compared with the age‐matched healthy control group (*n* = 42), the patient cohort showed substantially elevated leukocyte counts, NLR and CRP (Table 3). Median NLR was 2.79 (IQR 1.99–3.81) in patients versus 1.63 (1.19–2.29) in controls (*p* < 0.001); median CRP was 12.0 (5.45–25.5) versus 0.94 (0.10–4.20) mg/L (*p* < 0.001). Effect sizes were large for all three markers (Cliff's δ 0.49–0.68). Sensitivity analyses using the full control cohort (*n* = 74) yielded directionally identical results with similar effect magnitudes (Supporting Information S1: Table S1).

**TABLE 3:** Inflammatory biomarkers in patients versus age matched healthy controls.

Biomarker	Patients (*n* = 71) median (IQR)	Age‐matched controls (*n* = 42) median (IQR)	*p*‐value^a^	Cliff's *δ*
Leukocyte (× 10^3^/μL)	9.00 (7.55–11.20)	6.87 (6.05–8.83)	**< 0.001**	0.49 (large)
NLR	2.79 (1.99–3.81)	1.63 (1.19–2.29)	**< 0.001**	0.60 (large)
CRP (mg/L)	12.00 (5.45–25.50)	0.94 (0.10–4.20)	**< 0.001**	0.68 (large)

*Note*: Bold values in the p‐value column indicate statistically significant differences between patients and age‐matched controls (*p* < 0.05).

Abbreviations: CRP, C‐reactive protein; IQR, interquartile range; NLR, neutrophil‐to‐lymphocyte ratio.

^a^ Mann–Whitney *U* test. Sensitivity analysis with the full control group (*n* = 74) is provided in Supporting Information S1: Table S1.

### 3.4 Biomarkers by Clinical Features

Within the patient cohort (Table 4), NLR was significantly higher in patients with axillary lymphadenopathy (median 3.74 vs. 2.48, *p* = 0.022). Both NLR and CRP were significantly higher in patients with above mean lesion diameter (NLR *p* = 0.015; CRP *p* = 0.003). CRP was significantly higher in the surgical group (*p* = 0.008) and NLR showed a borderline difference (medical vs. surgical median NLR 2.26 vs. 2.92, *p* = 0.055). Leukocyte count did not differ across any of the examined subgroups.

**TABLE 4:** Association of inflammatory biomarkers with clinical features in IGM patients (*n* = 71).

Subgroup	*n*	NLR median (IQR)	*p*	CRP (mg/L) median (IQR)	*p*
Axillary LAP—absent	51	2.48 (1.99–3.35)	**0.022**	7.40 (4.98–24.00)	0.224
Axillary LAP—present	20	3.74 (2.12–5.15)		18.50 (6.71–33.25)	
Lesion ≤ 27.5 mm	41	2.43 (1.84–3.26)	**0.015**	7.00 (4.00–20.00)	**0.003**
Lesion > 27.5 mm	30	3.25 (2.27–4.71)		20.00 (7.47–29.75)	
Medical treatment	33	2.26 (1.82–3.26)	0.055	6.00 (3.50–20.00)	**0.008**
Surgical treatment	38	2.92 (2.21–4.37)		17.50 (7.06–28.75)	

*Note:* Mann–Whitney *U* test. Bold indicates *p* < 0.05. Leukocyte values omitted (no significant subgroup differences).

Abbreviation: LAP, lymphadenopathy.

### 3.5 Predictors of Surgical Treatment

On univariable logistic regression, only lesion diameter was a significant predictor of surgical treatment (OR 1.21 per mm; 95% CI 1.11–1.32; *p* < 0.001). NLR (OR 1.29; *p* = 0.086) and CRP (OR 1.03; *p* = 0.106) did not reach significance, nor did age, BMI or leukocyte count. Among clinical severity variables, none reached significance except bilateral disease, which showed an inverse association (OR 0.20; *p* = 0.022) suggesting that bilateral presentation was associated with lower odds of surgical treatment in this cohort. Fistula/ulcer, multi‐quadrant involvement, axillary lymphadenopathy, nipple retraction, erythema nodosum and ultrasonographic malignancy suspicion were not significant univariable predictors (all *p* > 0.20; Table 5).

**TABLE 5:** Univariable and multivariable logistic regression for surgical treatment.

Variable	OR	95% CI	*p*	aOR	95% CI	*p*
Age (per year)	0.99	0.94–1.04	0.683	0.98	0.90–1.05	0.526
BMI (per kg/m^2^)	1.06	0.97–1.16	0.223	—	—	—
Leukocyte (per 10^3^/μL)	1.09	0.92–1.30	0.311	—	—	—
NLR	1.29	0.96–1.73	0.086	0.81	0.49–1.34	0.414
CRP (per mg/L)	1.03	0.99–1.06	0.106	1.01	0.96–1.05	0.792
Lesion diameter (per mm)	**1.21**	**1.11–1.32**	**< 0.001**	**1.23**	**1.11–1.36**	**< 0.001**
Axillary LAP (present)	1.09	0.38–3.07	0.876	—	—	—

*Note:* Model 1 (a priori biomarker‐focused model): age + NLR + CRP + lesion diameter. Nagelkerke *R* ^2^ = 0.58; Hosmer–Lemeshow *p* = 0.37; AIC = 67.26. Model two (extended model incorporating clinical severity variables, added in response to reviewer request): age + NLR + CRP + lesion diameter + fistula/ulcer + multi‐quadrant involvement + axillary lymphadenopathy. Nagelkerke *R* ^2^ = 0.64; Hosmer–Lemeshow *p* = 0.51; AIC = 67.73. Bilateral disease, nipple retraction and erythema nodosum were not included in the multivariable models because of low event counts (*n* = 13, 3 and 4 respectively) that precluded stable estimation. US malignancy suspicion was collinear with several severity variables and was excluded from the a priori multivariable framework. Bold values indicate statistically significant associations (*p* < 0.05). This includes the entire “Lesion diameter” row (significant across univariable, Model 1 and Model 2 analyses) and the Model 2 estimates for axillary lymphadenopathy (*p* = 0.046).

Abbreviations: aOR, adjusted odds ratio; CI, confidence interval; CRP, C‐reactive protein; LAP, lymphadenopathy; NLR, neutrophil‐to‐lymphocyte ratio; OR, odds ratio; US, ultrasonography.

In the a priori biomarker‐focused multivariable model adjusting for age, NLR, CRP and lesion diameter, only lesion diameter retained independent predictive value (adjusted OR 1.23; 95% CI 1.11–1.36; *p* < 0.001). Neither NLR (aOR 0.81; *p* = 0.41) nor CRP (aOR 1.01; *p* = 0.79) contributed independently. Model fit was satisfactory (Nagelkerke *R* ^2^ = 0.58; Hosmer–Lemeshow *p* = 0.37) and the likelihood‐ratio test confirmed overall significance (*p* < 0.001).

To address the possibility that clinical severity variables might carry independent predictive information not captured by the biomarker‐focused model, we constructed an extended multivariable model adding fistula/ulcer, multi‐quadrant involvement and axillary lymphadenopathy (Table 5). Lesion diameter retained its independent predictive value with an effect size essentially unchanged from the biomarker‐focused model (aOR 1.25; 95% CI 1.13–1.40; *p* < 0.001). Fistula/ulcer (aOR 1.33; *p* = 0.77) and multi‐quadrant involvement (aOR 0.97; *p* = 0.97) did not contribute independently. Notably, axillary lymphadenopathy (which was not significant on univariable analysis (OR 1.09; *p* = 0.88)) reached borderline significance in the extended model in the inverse direction (aOR 0.08; 95% CI 0.01–0.96; *p* = 0.046). This directional reversal is a classical signature of confounding: axillary lymphadenopathy was correlated with lesion size (Table 4) and once lesion diameter is held constant, the residual variance in axillary lymphadenopathy pointed toward reduced rather than increased odds of surgical treatment. The clinical interpretation of this finding is developed in the Discussion. The extended model showed marginal improvement over the biomarker‐focused model in explanatory power (Nagelkerke *R* ^2^ = 0.64 vs. 0.58) but no meaningful improvement in fit (AIC 67.73 vs. 67.26; Hosmer–Lemeshow *p* = 0.51).

Two sensitivity analyses were performed to test whether these findings reflected the surgical group as a whole or were driven by drainage procedures (which comprised 79% of surgical cases). When the surgical group was restricted to drainage versus medical treatment only (*n* = 63), lesion diameter remained the sole independent predictor with a comparable effect size (aOR 1.27; 95% CI 1.13–1.43; *p* < 0.001; Supporting Information S1: Table S2). When the surgical group was restricted to excisional procedures versus medical treatment (*n* = 41), lesion diameter remained a significant univariable predictor with a smaller effect size (OR 1.12 per mm; 95% CI 1.03–1.21; *p* = 0.006); in this subgroup NLR reached borderline univariable significance (OR 1.48; 95% CI 1.01–2.18; *p* = 0.046)—a finding not observed in the primary analysis and requiring cautious interpretation given the small event count of 8 (Supporting Information S1: Table S3).

### 3.6 Diagnostic Performance for Treatment Selection

Lesion diameter alone discriminated medical from surgical treatment with high accuracy (AUC 0.914, 95% CI 0.833–0.978; optimal cut‐off 22 mm: sensitivity 92.1%, specificity 84.8%; Table 6, Figure 2). NLR (AUC 0.633, 95% CI 0.500–0.760) and CRP (AUC 0.683, 95% CI 0.555–0.809) showed only modest discrimination, with bootstrap confidence intervals approaching the non‐informative threshold of 0.5. Leukocyte count was not discriminative (AUC 0.571).

**TABLE 6:** ROC analysis for predicting surgical treatment (single biomarkers, biomarker combinations and full extended model).

Predictor	AUC	95% CI (bootstrap)	Cut‐off	Sens	Spec
Leukocyte	0.571	0.432–0.701	10.40 × 10^3^/μL	0.47	0.76
NLR	0.633	0.500–0.760	2.27	0.74	0.52
CRP	0.683	0.555–0.809	7.05 mg/L	0.76	0.58
Lesion diameter	**0.914**	**0.833–0.978**	**22 mm**	**0.92**	**0.85**
NLR + CRP	0.694	0.552–0.816	—	0.79	0.61
NLR + lesion diameter	0.908	0.821–0.976	—	0.92	0.85
CRP + lesion diameter	0.912	0.830–0.979	—	0.92	0.85
NLR + CRP + lesion diameter	0.911	0.825–0.978	—	0.92	0.85
*Full extended model* *	*0.918*	*0.852–0.974*	—	*0.87*	*0.85*

*Note*: Bold values highlight the results for lesion diameter, which represents the primary predictor of interest and the variable most strongly associated with treatment selection in this study.

Abbreviations: AUC, area under the receiver operating characteristic curve; CRP, C‐reactive protein; NLR, neutrophil‐to‐lymphocyte ratio; Spec, specificity; Sens, sensitivity.

^*^ Full extended model: age + NLR + CRP + lesion diameter + fistula/ulcer + multi‐quadrant involvement + axillary lymphadenopathy. 95% confidence intervals were derived from 1000 bootstrap resamples. Pairwise DeLong tests for AUC comparisons: three‐variable biomarker model versus lesion diameter alone, ΔAUC = −0.003, *p* = 0.67; full extended model versus lesion diameter alone, ΔAUC = +0.004, *p* = 0.89; full extended model versus three‐variable biomarker model, ΔAUC = +0.006, *p* = 0.25. Sens/Spec for combined models were derived from the Youden index applied to the predicted probability of the multivariable model.

**FIGURE 2:**
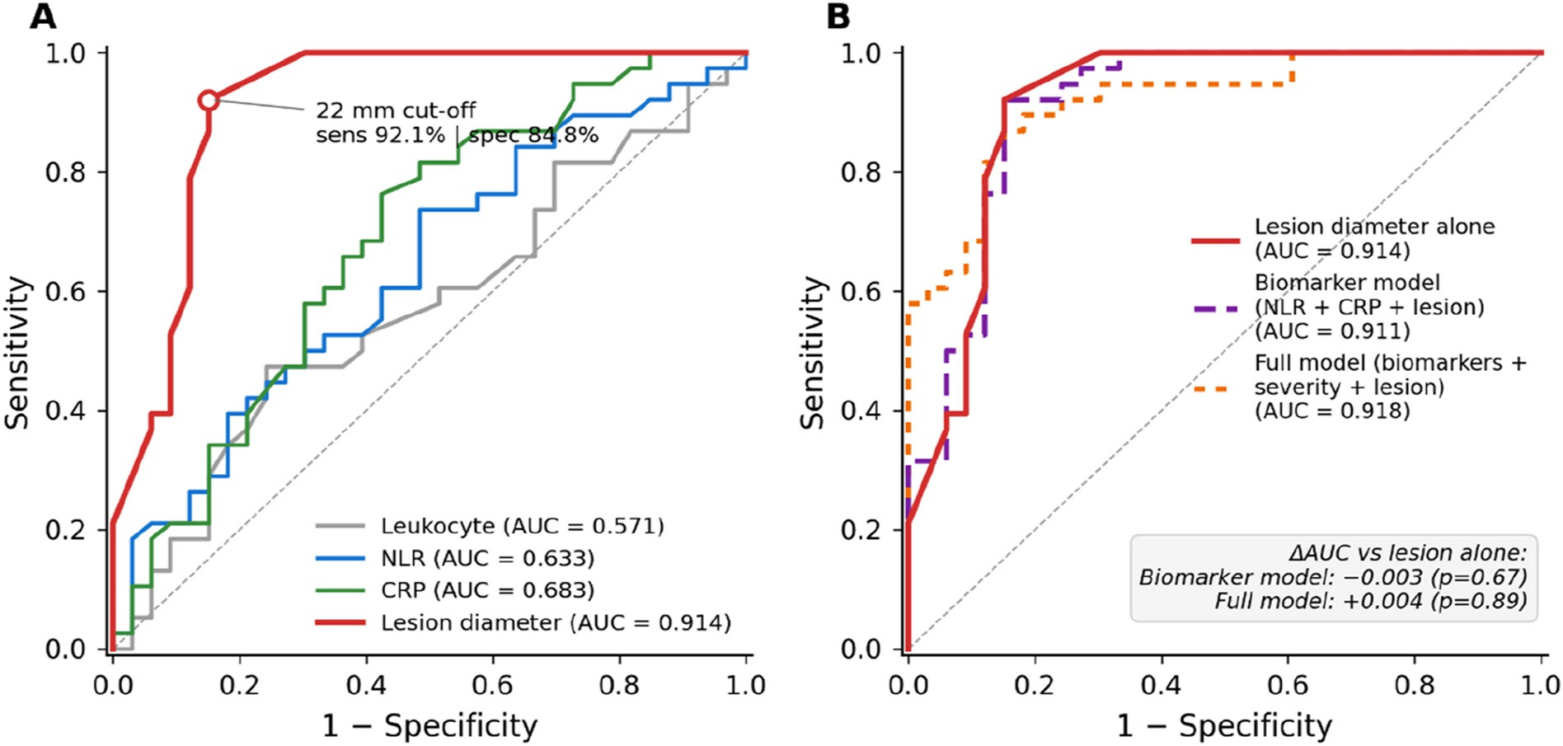
Receiver operating characteristic (ROC) curves for the prediction of surgical treatment in idiopathic granulomatous mastitis (*n* = 71). (A) Discriminative performance of single candidate biomarkers (leukocyte count, neutrophil‐to‐lymphocyte ratio [NLR], C‐reactive protein [CRP]) compared with ultrasonographic lesion diameter. Lesion diameter showed substantially higher discrimination (AUC 0.914) than inflammatory biomarkers (AUC 0.571–0.683). The optimal cut‐off for lesion diameter, identified by the Youden index at 22 mm, is indicated on the curve. (B) Comparison of lesion diameter alone (AUC 0.914) with the biomarker‐focused model (NLR + CRP + lesion diameter; AUC 0.911) and the extended multivariable model incorporating clinical severity variables (age + NLR + CRP + lesion diameter + fistula/ulcer + multi‐quadrant involvement + axillary lymphadenopathy; AUC 0.918). Pairwise DeLong tests: biomarker‐focused model versus lesion diameter alone, ΔAUC = −0.003, *p* = 0.67; extended model versus lesion diameter alone, ΔAUC = +0.004, *p* = 0.89.

Biomarker combinations did not improve on lesion diameter alone (Table 6). The three‐variable biomarker combination (NLR + CRP + lesion diameter; AUC 0.911) was not significantly different from lesion diameter alone (ΔAUC −0.003; DeLong test *p* = 0.67). Two‐variable combinations performed similarly (NLR + lesion diameter AUC 0.910; CRP + lesion diameter AUC 0.913). The combination of NLR and CRP without lesion diameter performed substantially worse (AUC 0.695). Consistent with the extended multivariable regression results, incorporating clinical severity variables did not improve discrimination either: the full model incorporating biomarkers, severity variables and lesion diameter yielded AUC 0.918, which was not significantly different from lesion diameter alone (ΔAUC +0.004; DeLong test *p* = 0.89; Figure 2).

### 3.7 Recurrence

In exploratory analyses, no biomarker discriminated recurrent from non recurrent disease (all *p* > 0.05; Supporting Information S1: Table S4). Median pretreatment values in recurrent versus non recurrent cases were as follows: NLR 1.90 versus 2.80 (*p* = 0.19), CRP 16.50 versus 10.00 mg/L (*p* = 0.55), leukocyte 8.90 versus 9.00 × 10^3^/μL (*p* = 0.88). Given only 12 recurrence events, these analyses were substantially underpowered.

## 4 Discussion

The principal finding of this study is that, in patients with biopsy‐proven idiopathic granulomatous mastitis, ultrasonographic lesion diameter is the dominant independent predictor of treatment modality after adjustment for standard inflammatory biomarkers and standard clinical severity variables. Neither NLR nor CRP and neither fistula/ulcer formation, multi‐quadrant involvement nor axillary lymphadenopathy contributed independent predictive value once lesion size was taken into account. Biomarker combinations, severity‐only combinations and full multivariable combinations all failed to improve on lesion diameter alone in ROC analysis. These findings should be interpreted as characterizing the pattern of treatment selection in this cohort rather than as an intrinsic hierarchy of disease‐severity markers; this distinction is developed below.

This result reframes the role of inflammatory biomarkers in IGM clinical decision making. Previous studies, including those of Çetinkaya et al. [8] and Li et al. [9], have proposed NLR as a clinically useful predictor in IGM. However, both reports focused on recurrence prediction rather than initial treatment selection and neither adjusted for lesion morphology in their multivariable models. A more recent investigation by Sağlık et al. [12] specifically tested CRP, NLR and other hematological indices for recurrence prediction in 92 IGM patients and found no significant association, in concordance with our exploratory recurrence findings. Velidedeoğlu et al. [13] reported high diagnostic sensitivity for CRP in distinguishing IGM from breast cancer and Yabanoğlu et al. [11] showed similar utility against breast abscess; both addressed diagnostic differentiation rather than therapeutic stratification. To our knowledge, no prior study of IGM has directly examined the incremental predictive value of inflammatory biomarkers beyond lesion diameter for the medical‐versus‐surgical decision and no prior study has compared these biomarkers against a set of standard clinical severity variables within the same multivariable framework. Our results indicate that this incremental value is negligible for both categories of variable: neither inflammatory biomarkers nor standard clinical severity markers add discriminative or independent predictive information beyond that already carried by lesion diameter.

Three complementary observations emerge regarding the biology of the studied markers. First, NLR and CRP correlated meaningfully with lesion burden in univariable comparisons: both were significantly higher in patients with above‐mean lesion diameter and CRP was significantly higher in the surgical group (Table 4). This pattern is consistent with a role for these biomarkers as reflections of inflammatory activity and disease extent, in line with their broader use in inflammatory conditions [10, 13]. However, this correlation is conceptually distinct from independent predictive value. Once lesion size is entered into the multivariable framework, the additional information carried by NLR and CRP becomes statistically negligible, indicating that these biomarkers are largely informationally redundant with lesion morphology as measured by ultrasound. The clinical implication is that NLR and CRP may still be useful as markers of inflammatory burden (e.g., in monitoring response to medical treatment or in flagging patients with high inflammatory activity) but should not be used in isolation to determine treatment modality. Second, the substantial elevation of NLR, CRP and leukocyte count in IGM patients relative to age‐matched healthy controls (with large effect sizes for all three) confirms the systemic inflammatory nature of IGM and supports biomarker utility for diagnostic or differential‐diagnostic purposes [10, 13, 14]. The findings of the present study argue against extending that diagnostic utility to therapeutic decision‐making without adjustment for lesion morphology; they do not negate the diagnostic role itself. Third, axillary lymphadenopathy (which was not a univariable predictor of surgical treatment) reached borderline significance in the extended multivariable model in the inverse direction (aOR 0.08; *p* = 0.046). Because the direction of this association was reversed by multivariable adjustment, the finding is best understood as an example of confounding by lesion size rather than as a genuine protective effect of axillary lymphadenopathy. Axillary lymphadenopathy was correlated with lesion diameter (Table 4), and once lesion size was held constant the residual variance in axillary lymphadenopathy no longer pointed in the direction one would expect from a marker of disease burden. A cautious clinical interpretation is that among patients matched for lesion size, those presenting with axillary lymphadenopathy may reflect a reactive systemic inflammatory response for which the treating surgeon opted for medical rather than surgical management in the expectation of a favorable response to corticosteroid therapy. This interpretation is hypothetical, based on a single borderline result and requires prospective validation before it can be taken as evidence of a genuine mechanism.

A methodological strength of this study is the explicit comparison with an age matched control group. Inflammatory biomarkers, particularly NLR, vary systematically with age; published reference values range from approximately 1.65 in younger populations to over 2.10 in older U.S. cohorts [15, 16]. Many prior IGM studies have compared young female patients with older or mixed age controls, potentially inflating apparent group differences. Restricting comparison to controls aged 18–50—matched on both age and BMI—addresses this confounding directly and strengthens the inference that IGM‐associated biomarker elevations are disease related rather than age‐related. A second methodological contribution is the use of bootstrap derived confidence intervals together with DeLong's test for AUC comparisons. Earlier reports of very high AUC values for biomarker combinations in IGM have, in some cases, relied on predicted probabilities from the same regression model evaluated against the data that generated them—a procedure that can give the impression of additive predictive value where none exists—without formal statistical testing for incremental discrimination. When AUC differences are tested directly with DeLong's method, the genuine incremental value of NLR and CRP beyond lesion diameter approaches zero. A third methodological contribution, added in response to peer review, was the construction of an extended multivariable model incorporating standard clinical severity variables (fistula/ulcer formation, multi‐quadrant involvement and axillary lymphadenopathy) alongside inflammatory biomarkers and lesion diameter. This extended model addresses the concern that observed predictors might be reduced or overturned once severity markers are accounted for. The independent predictive value of lesion diameter was essentially unchanged (aOR 1.25 vs. 1.23) and no severity variable reached independent significance in the expected direction. A fourth methodological element was the use of sensitivity analyses restricting the surgical group to drainage only or to excisional procedures only; these confirmed that the observed association between lesion diameter and treatment selection was not driven by one surgical subgroup.

The clinical implication is that under the pattern of treatment selection observed in this cohort, ultrasonographic lesion diameter (readily available, inexpensive and operator‐independent at the level of mm‐scale measurement) was the variable most strongly associated with the medical‐versus‐surgical decision. At a cut‐off of 22 mm, lesion diameter achieved sensitivity of 92.1% and specificity of 84.8% for predicting surgical treatment. The sensitivity analyses restricted to drainage and to excisional procedures separately support the robustness of this association across surgical subgroups, although with a smaller effect size in the excisional subgroup where the number of events was limited. It should be emphasized that these findings characterize the pattern of treatment selection within the studied cohort rather than establishing lesion diameter as an intrinsic marker of disease severity; the retrospective design and the absence of a protocolized decision algorithm mean that lesion size may have been weighted heavily by the treating surgeon a priori and prospective validation with a predefined treatment protocol is essential before the 22 mm threshold can be recommended as a standalone clinical anchor. Inflammatory biomarkers may complement clinical assessment as markers of disease activity, particularly in monitoring or in atypical presentations, but they should not be used in isolation to determine treatment modality. The exploratory recurrence analyses did not identify any biomarker with statistically significant predictive value. Numerically, NLR was somewhat lower in recurrent than in non recurrent cases (median 1.90 vs. 2.80), while CRP showed the opposite direction (16.50 vs. 10.00 mg/L); neither difference approached significance. Although the analysis was substantially underpowered with only 12 events, the heterogeneous directionality of these markers—together with the negative findings of Sağlık et al. [12]—argues against a simple inflammatory‐burden model of recurrence prediction in IGM.

### 4.1 Limitations

Several limitations should be acknowledged. First, the retrospective single center design restricts generalizability and introduces the possibility of selection and information bias. Second, the treatment decision was made by the attending surgeon without a standardized algorithm; no written institutional protocol prescribed the choice between drainage, wide local excision and segmentectomy for surgical patients. While the retrospective reconstruction of typical decision patterns suggested consistent tendencies (drainage for abscess or fluctuant collection, wide local excision for surgically resectable lesions, segmentectomy for segmental disease), individual clinical judgment inevitably introduced variability that we could not fully quantify. Lesion diameter may have been weighted heavily a priori by the treating surgeon, which would bias toward a strong observed association between lesion size and surgical treatment. The pattern of association reported here should therefore be understood as a description of how treatment was selected in this cohort rather than as a normative criterion for how it should be selected, and prospective validation in cohorts using a predefined treatment protocol is essential. Third, the recurrence analysis was underpowered with only 12 events and should be regarded as exploratory; larger multicenter cohorts are needed to evaluate biomarker performance for recurrence prediction. Fourth, additional inflammatory or autoimmune markers (interleukin‐33, ST2, HLA typing, albumin‐to‐globulin ratio) that may carry independent information were not assessed. Fifth, polymerase chain reaction examination of tissue specimens to exclude occult specific infections was not routinely performed. Finally, although the age matched control group represents a methodological strength, healthy women undergoing elective hernia repair may not be perfectly representative of the general population and minor residual confounding cannot be excluded.

## 5 Conclusion

In this cohort of patients with biopsy‐proven idiopathic granulomatous mastitis, serum NLR and CRP were markedly elevated relative to age‐matched healthy controls and correlated with lesion burden, supporting their role as markers of inflammatory activity and disease extent. However, neither NLR and CRP nor standard clinical severity variables (fistula/ulcer formation, multi‐quadrant involvement and axillary lymphadenopathy) added independent predictive value beyond ultrasonographic lesion diameter for the medical‐versus‐surgical decision. Lesion diameter was the variable most strongly associated with treatment selection in this cohort, with strong discriminative performance (AUC 0.91; 22 mm cut‐off). These findings should be interpreted as characterizing the pattern of treatment selection under the observed clinical decision‐making rather than as an intrinsic hierarchy of disease‐severity markers. Inflammatory biomarkers may retain a role as markers of inflammatory burden and disease activity in clinical monitoring but should not be relied upon in isolation to determine treatment modality. Prospective multicenter cohorts with predefined treatment algorithms are needed to validate the 22 mm threshold, to test whether additional variables carry independent predictive information under protocolized decision‐making and to examine biomarker performance for recurrence prediction in adequately powered event samples.

## Author Contributions

**Muhammet Fatih Keyif:** conceptualization, methodology, formal analysis, investigation, visualization, writing – original draft, writing – review and editing. **Sabahattin Destek:** conceptualization, methodology, data curation, resources, supervision, project administration, writing – review and editing.

## Funding

The authors have nothing to report.

## Ethics Statement

This study was approved by the Ethics Committee of Bezmialem Vakıf University Faculty of Medicine (decision number 16/299, dated 10 September 2019), covering the analysis of inflammatory biomarkers (NLR and CRP) in idiopathic granulomatous mastitis. The present manuscript represents a secondary analysis of the cohort approved under this decision, examining treatment modality prediction rather than diagnostic prediction. The research was conducted in accordance with the principles of the Declaration of Helsinki. Given the retrospective design and use of de‐identified clinical records, the requirement for individual informed consent was waived by the Ethics Committee.

## Conflicts of Interest

The authors declare no conflicts of interest.

## Supporting information


Supporting Information S1

## Data Availability

The data that support the findings of this study are available from the corresponding author upon reasonable request. The data are not publicly available due to privacy and ethical restrictions related to patient health information.

## References

[1] M. M. Baslaim , H. A. Khayat , S. A. Al‐Amoudi , and H. K. Mohamed , “Idiopathic Granulomatous Mastitis: A Heterogeneous Disease With Variable Clinical Presentation,” World Journal of Surgery 31, no. 8 (2007): 1677–1681, 10.1007/s00268-007-9116-1. 17541683

[2] A. Wolfrum , S. Kümmel , I. Theuerkauf , E. Pelz , and M. Reinisch , “Granulomatous Mastitis: A Therapeutic and Diagnostic Challenge,” Breast Care 13, no. 6 (2018): 413–418, 10.1159/000495146. PMC6381909 30800035

[3] B. B. Ozcan , K. Merchant , J. Goldberg , et al., “Can Imaging Findings Predict the Outcome of Idiopathic Granulomatous Mastitis?,” Annals of Breast Surgery 7 (2023): 35, 10.21037/abs-22-23.

[4] D. Martinez‐Ramos , L. Simon‐Monterde , C. Suelves‐Piqueres , et al., “Idiopathic Granulomatous Mastitis: A Systematic Review of 3060 Patients,” Breast Journal 25, no. 6 (2019): 1245–1250, 10.1111/tbj.13446. 31273861

[5] M. Emiroglu , A. Akcan , M. Velidedeoglu , et al., “Diagnosis, Approach, and Clinical Classification of Idiopathic Granulomatous Mastitis: Consensus Report,” Breast Care 19, no. 5 (2024): 243–251, 10.1159/000540547. PMC11493389 39439860

[6] C. M. Freeman , B. T. Xia , G. C. Wilson , et al., “Idiopathic Granulomatous Mastitis: A Diagnostic and Therapeutic Challenge,” American Journal of Surgery 214, no. 4 (2017): 701–706, 10.1016/j.amjsurg.2017.07.002. 28739122

[7] K. Çetin , H. E. Sıkar , N. E. Göret , et al., “Comparison of Topical, Systemic, and Combined Therapy With Steroids on Idiopathic Granulomatous Mastitis: A Prospective Randomized Study,” World Journal of Surgery 43, no. 11 (2019): 2865–2873, 10.1007/s00268-019-05084-x. 31297582

[8] Ö. A. Çetinkaya , S. U. Çelik , S. G. Terzioğlu , and A. Eroğlu , “The Predictive Value of the Neutrophil‐To‐Lymphocyte and Platelet‐To‐Lymphocyte Ratio in Patients With Recurrent Idiopathic Granulomatous Mastitis,” European Journal of Breast Health 16, no. 1 (2020): 61–65, 10.5152/ejbh.2019.5187. PMC6939709 31912016

[9] Q. Li , J. Wan , Z. Feng , J. Shi , and W. Wei , “Predictive Significance of the Preoperative Neutrophil‐Lymphocyte Ratio for Recurrence in Idiopathic Granulomatous Mastitis Patients,” American Surgeon 89, no. 12 (2023): 5577–5583, 10.1177/00031348231161793. 36880848

[10] X. Wang , X. He , J. Liu , et al., “Immune Pathogenesis of Idiopathic Granulomatous Mastitis: From Etiology Toward Therapeutic Approaches,” Frontiers in Immunology 15 (2024): 1295759, 10.3389/fimmu.2024.1295759. PMC10961981 38529282

[11] H. Yabanoğlu , H. Akkoç , H. Köksal , et al., “White Blood Count Can Be a Practical Guide for the Differential Diagnosis of Breast Abscess and Idiopathic Granulomatous Mastitis,” Cureus 12, no. 9 (2020): e10468, 10.7759/cureus.10468. PMC7566977 33083171

[12] S. Sağlık , “The Role of Clinical and Radiological Risk Factors and Triglyceride‐Glucose Index in Predicting Recurrence of Idiopathic Granulomatous Mastitis,” Journal of Medicine and Palliative Care 5, no. 5 (2024): 473–478, 10.47582/jompac.1539100.

[13] M. Velidedeoğlu , B. Papila Kundaktepe , H. Aksan , and H. Uzun , “Preoperative Fibrinogen and Hematological Indexes in the Differential Diagnosis of Idiopathic Granulomatous Mastitis and Breast Cancer,” Medicina 57, no. 7 (2021): 698, 10.3390/medicina57070698. PMC8303264 34356979

[14] H. Koksal , H. Vatansev , H. Artac , and N. Kadoglou , “The Clinical Value of Interleukins‐8, ‐10, and ‐17 in Idiopathic Granulomatous Mastitis,” Clinical Rheumatology 39, no. 5 (2020): 1671–1677, 10.1007/s10067-020-04925-8. 31916110

[15] J. S. Lee , N. Y. Kim , S. H. Na , Y. H. Youn , and C. S. Shin , “Reference Values of Neutrophil‐Lymphocyte Ratio, Lymphocyte‐Monocyte Ratio, Platelet‐Lymphocyte Ratio, and Mean Platelet Volume in Healthy Adults in South Korea,” Medicine 97, no. 26 (2018): e11138, 10.1097/MD.0000000000011138. PMC6039688 29952958

[16] B. Azab , M. Camacho‐Rivera , and E. Taioli , “Average Values and Racial Differences of Neutrophil Lymphocyte Ratio Among a Nationally Representative Sample of United States Subjects,” PLoS One 9, no. 11 (2014): e112361, 10.1371/journal.pone.0112361. PMC4223021 25375150

