# Supplementary material for: Lesion Diameter Versus Inflammatory Biomarkers and Clinical Severity in Predicting Treatment Selection in Idiopathic Granulomatous Mastitis
Source: World J Surg. 2026 Aug 31;50(10):2817–27. doi: 10.1002/wjs.70554 (PMC13662512; doi:10.1002/wjs.70554)
Supplement: Supplementary file 1 [file WJS-50-2817-s001.docx]

**Supplementary Figure S1.** STROBE flow diagram of patient selection. Of 92 women with a clinical and radiological preliminary diagnosis of idiopathic granulomatous mastitis (IGM) presenting between January 2015 and December 2019, 21 were excluded for the reasons indicated, leaving 71 patients with biopsy-proven IGM as the final analytic cohort. Patients were grouped by treatment modality (medical only vs any surgical procedure) for the primary outcome analysis.

**Supplementary Table S1.** Inflammatory biomarkers in IGM patients versus the full healthy control group (sensitivity analysis)

*This table presents the sensitivity analysis using all 74 healthy controls (without age restriction), corresponding to the primary analysis shown in Table 3 of the main manuscript (which used the age-matched subset, n = 42).*

| **Biomarker** | **Patients (n = 71) median (IQR)** | **All controls (n = 74) median (IQR)** | **p-value*** | **Cliff's δ** |
| --- | --- | --- | --- | --- |
| Leukocyte (×10³/µL) | 9.00 (7.55–11.20) | 7.08 (6.08–8.66) | **<0.001** | 0.49 (large) |
| NLR | 2.79 (1.99–3.81) | 1.60 (1.20–2.22) | **<0.001** | 0.61 (large) |
| CRP (mg/L) | 12.00 (5.45–25.50) | 0.97 (0.21–3.98) | **<0.001** | 0.74 (large) |

**Mann–Whitney U test. NLR: neutrophil-to-lymphocyte ratio; CRP: C-reactive protein; IQR: interquartile range. The full control cohort (n = 74) had a mean age of 48.7 ± 15.7 years (range 22–79), which exceeded the patient cohort age distribution (37.0 ± 8.8 years). Despite this age disparity, between group differences in all three biomarkers remained highly significant with large effect sizes, indicating that the elevations observed in IGM patients are not attributable to age related variation alone. These directionally identical findings support the robustness of the primary analysis presented in Table 3.*

**Supplementary Table S2.** Sensitivity analysis: drainage (n = 30) versus medical treatment only (n = 33)

| **Variable** | **Univariable OR**  **(95% CI)** | **p** | **Multivariable* aOR**  **(95% CI)** | **p** |
| --- | --- | --- | --- | --- |
| Age (per year) | — | — | 1.00 (0.89–1.12) | 0.993 |
| NLR | 1.22 (0.89–1.66) | 0.219 | 0.92 (0.51–1.67) | 0.782 |
| CRP (per mg/L) | 1.03 (1.00–1.06) | 0.075 | 1.01 (0.97–1.06) | 0.569 |
| **Lesion diameter (per mm)** | **1.23 (1.11–1.35)** | **<0.001** | **1.27 (1.13–1.43)** | **<0.001** |
| Fistula/ulcer (present) | 0.93 (0.27–3.16) | 0.906 | 1.72 (0.22–13.67) | 0.610 |
| Multi-quadrant involvement | 1.34 (0.44–4.08) | 0.608 | 0.94 (0.12–7.75) | 0.958 |
| Axillary LAP | 1.14 (0.38–3.41) | 0.811 | 0.10 (0.01–1.59) | 0.103 |

**Multivariable model: age + NLR + CRP + lesion diameter + fistula/ulcer + multi-quadrant involvement + axillary lymphadenopathy. Nagelkerke R² = 0.67; AIC = 59.13; area under the ROC curve for lesion diameter = 0.923. This sensitivity analysis addresses the question of whether the observed predictors reflect surgical treatment as a whole or primarily the need for drainage. Lesion diameter retained independent predictive value with an effect size similar to the primary analysis. OR: odds ratio; aOR: adjusted odds ratio; NLR: neutrophil-to-lymphocyte ratio; CRP: C-reactive protein; LAP: lymphadenopathy.*

**Supplementary Table S3.** Sensitivity analysis: excisional surgery (n = 8) versus medical treatment only (n = 33)

| **Variable** | **Univariable OR (95% CI)** | **p** | **Multivariable† aOR**  **(95% CI)** | **p** |
| --- | --- | --- | --- | --- |
| **Lesion diameter (per mm)** | **1.12 (1.03–1.21)** | **0.006** | **1.12 (1.03–1.21)** | **0.010** |
| **NLR** | **1.48 (1.01–2.18)** | **0.046** | — | — |
| CRP (per mg/L) | 1.00 (0.96–1.05) | 0.913 | — | — |
| Fistula/ulcer (present) | 0.53 (0.06–5.06) | 0.582 | 0.88 (0.08–9.92) | 0.917 |
| Multi-quadrant involvement | 1.87 (0.36–9.65) | 0.452 | 0.98 (0.13–7.62) | 0.986 |
| Axillary LAP | 0.89 (0.15–5.24) | 0.897 | — | — |

*†Parsimonious multivariable model (lesion diameter + fistula/ulcer + multi-quadrant involvement) selected because of the limited number of events (n = 8) precluded larger models. This sensitivity analysis restricts the surgical group to wide local excision and segmentectomy, excluding drainage procedures. Lesion diameter retained independent predictive value albeit with a smaller effect size than in the drainage-inclusive analysis. In this subgroup, NLR reached borderline univariable significance (OR 1.48, p = 0.046), which was not observed in the primary analysis; this finding should be interpreted cautiously given the small sample size and is discussed in the manuscript. Area under the ROC curve for lesion diameter = 0.881. OR: odds ratio; aOR: adjusted odds ratio; NLR: neutrophil-to-lymphocyte ratio; CRP: C-reactive protein; LAP: lymphadenopathy*

**Supplementary Table S4.** Exploratory analysis of factors associated with disease recurrence

*This exploratory subgroup analysis compares baseline characteristics, inflammatory biomarkers and treatment between patients who experienced recurrence (n = 12, 16.9%) during follow-up and those who did not (n = 59). Analyses were prespecified as exploratory given the limited number of recurrence events.*

| **Variable** | **Recurrent (n = 12)** | **Non-recurrent (n = 59)** | **p-value*** | **Test** |
| --- | --- | --- | --- | --- |
| Age, years | 37.5 (35.5–42.0) | 36.0 (30.5–41.5) | 0.369 | MW |
| BMI, kg/m² | 26.17 (24.73–29.06) | 26.54 (22.38–30.24) | 0.854 | MW |
| Lesion diameter, mm | 31.5 (20.0–54.0) | 24.0 (16.0–34.5) | 0.091 | MW |
| Leukocyte, ×10³/µL | 8.90 (7.38–10.50) | 9.00 (7.60–11.20) | 0.884 | MW |
| NLR | 1.90 (1.50–3.53) | 2.80 (2.16–3.81) | 0.192 | MW |
| CRP, mg/L | 16.50 (6.36–23.75) | 10.00 (5.45–25.50) | 0.549 | MW |
| Surgical treatment, n (%) | 7 (58.3) | 31 (52.5) | 0.761 | Fisher |
| Axillary LAP, n (%) | 5 (41.7) | 15 (25.4) | 0.299 | Fisher |

*Continuous variables expressed as median (interquartile range). *MW: Mann–Whitney U test; Fisher: Fisher's exact test. NLR: neutrophil-to-lymphocyte ratio; CRP: C-reactive protein; LAP: lymphadenopathy. No variable reached statistical significance. With only 12 recurrence events, this analysis was substantially underpowered; results should be interpreted with caution, particularly for lesion diameter (p = 0.091), which approached but did not reach the conventional significance threshold.*
